# Genome-wide identification of potato long intergenic noncoding RNAs responsive to *Pectobacterium carotovorum* subspecies *brasiliense* infection

**DOI:** 10.1186/s12864-016-2967-9

**Published:** 2016-08-11

**Authors:** Stanford Kwenda, Paul R. J. Birch, Lucy N. Moleleki

**Affiliations:** 1 Forestry and Agricultural Biotechnology Institute (FABI), Genomics Research Institute (GRI), Department of Microbiology and Plant Pathology, University of Pretoria, Pretoria, 0028 South Africa; 2 The Division of Plant Sciences, College of Life Sciences, University of Dundee (at The James Hutton Institute), Dundee, DD25DA Scotland, UK

**Keywords:** Noncoding RNA, Potato, *Pectobacterium*, lincRNA, Plant defence, Soft rot bacteria, *Solanum tuberosum*, Entereobacteria

## Abstract

**Background:**

Long noncoding RNAs (lncRNAs) represent a class of RNA molecules that are implicated in regulation of gene expression in both mammals and plants. While much progress has been made in determining the biological functions of lncRNAs in mammals, the functional roles of lncRNAs in plants are still poorly understood. Specifically, the roles of long intergenic nocoding RNAs (lincRNAs) in plant defence responses are yet to be fully explored.

**Results:**

In this study, we used strand-specific RNA sequencing to identify 1113 lincRNAs in potato (*Solanum tuberosum*) from stem tissues. The lincRNAs are expressed from all 12 potato chromosomes and generally smaller in size compared to protein-coding genes. Like in other plants, most potato lincRNAs possess single exons. A time-course RNA-seq analysis between a tolerant and a susceptible potato cultivar showed that 559 lincRNAs are responsive to *Pectobacterium carotovorum* subsp. *brasiliense* challenge compared to mock-inoculated controls. Moreover, coexpression analysis revealed that 17 of these lincRNAs are highly associated with 12 potato defence-related genes.

**Conclusions:**

Together, these results suggest that lincRNAs have potential functional roles in potato defence responses. Furthermore, this work provides the first library of potato lincRNAs and a set of novel lincRNAs implicated in potato defences against *P. carotovorum* subsp. *brasiliense*, a member of the soft rot *Enterobacteriaceae* phytopathogens.

**Electronic supplementary material:**

The online version of this article (doi:10.1186/s12864-016-2967-9) contains supplementary material, which is available to authorized users.

## Background

Advances in transcriptome profiling techniques especially with the advent of deep sequencing approaches (RNA-sequencing) have revealed that transcription in eukaryotes is much more complex than previously anticipated. It is now apparent that the bulk of eukaryotic genomes is pervasively transcribed giving rise to noncoding RNAs (ncRNAs) which exert pivotal effects on gene regulation [1]. Noncoding RNAs can be grouped based on their lengths, into either (1) short ncRNAs (<200 bp) which have been extensively studied and generally include microRNAs (miRNAs), small nucleolar RNAs (snoRNAs), small nuclear RNA (snRNAs), and small interfering RNAs (siRNAs); and (2) long ncRNAs which are generally greater than 200 bp in length. Like mRNAs, lncRNAs have a 5’ cap and a 3’ poly-A tail; are mostly localized within the nucleus [2, 3], and can be multi-exonic [4]. LncRNAs can exhibit cell or tissue specific expression patterns and have been observed to show poor conservation across different species [4]. Based on their genomic location and context, lncRNAs are classified into intergenic (long intergenic noncoding RNA; lincRNA), long intronic noncoding RNA, and natural antisense transcripts (NATs). Natural antisense transcripts are RNA molecules with complementarity to other transcripts and can be grouped into *cis*-NATs (NATs fully antisense to protein coding genes on opposite strand) and *trans*-NATs (NATs with partial complementarity and transcribed from different loci) [1]. Some lincRNAs can be located in close proximity to protein-coding genes (CDS), thus, may be referred to as adjacent-lncRNAs, and usually associated with CDS promoter and terminator regions. Furthermore, lincRNAs on one strand can partially overlap with CDS regions on the opposite strand and such lincRNAs may be termed antisense-lncRNAs.

In the past decade, much progress has been made towards understanding the roles of small non coding RNAs in plants [5]. However, unlike small RNAs, the regulatory roles of lncRNAs remain poorly understood. Furthermore, compared to human and animal species, genome-wide discovery of lncRNAs in plants is still in its infancy [6]. Consequently, lncRNAs in plants constitute a class of ncRNAs that is less well-characterized. Nonetheless, regulatory roles of plant lncRNAs are now beginning to be recognized in diverse plant species through employing whole genome tilling arrays, *in silico* predictions and RNA-seq approaches [7–11]. These emerging evidences demonstrate that lncRNAs play important roles in diverse biological processes in plants ranging from plant reproductive development, and responses to biotic and abiotic stresses [9, 12, 13].

The functional mechanisms of lncRNAs in many plant species are not yet fully understood with only a few lncRNAs having been fully characterized. In Arabidopsis, lncRNAs such as COLDAIR (cold-assisted intronic non-coding RNA) and COOLAIR (cold induced long antisense intragenic RNA) have been demonstrated to mediate chromatin modifying activities in transcriptional silencing of *FLC* during vernalization [14, 15]. Another antisense lncRNA, ASL, a non-polyadenylated transcript, was recently discovered, and is implicated in epigenetic silencing of *FLC* [16]. Additional regulatory functions of some lincRNAs such as *AT4* and *IPS1* (*INDUCED BY PHOSPHATE STARVATION1*) involve acting as decoys of miRNAs by a target mimicry mechanism, thus sequestering the regulatory roles of miRNAs away from their intended target genes [17–19]. It has recently been suggested that the Alternative Splicing Competitor long noncoding RNA (*ASCO-*lncRNA) also acts as a decoy, regulating gene expression in Arabidopsis during development [20]. The *ASCO*-lncRNA acts by competing to bind alternative splicing (AS) regulators, thus, diverting them from their AS mRNA targets [20]. Furthermore, plant lincRNAs have been implicated in important biological roles in responses to external stimuli [18, 19, 21, 22]. In plants, genome-wide analysis of lncRNAs using deep sequencing transcriptomic data (mainly from RNA-seq approaches) have been performed on only a few plant species including *Arabidopsis thaliana* [9, 13, 23], *Triticum aestivum* [8], *Medicago truncatula* [24], *Oryza sativa* [12] tomato [25] and *Zea mays* [10, 26]. Recently, a computational genome scale investigation of lncRNAs associated with annotated gene models was performed on 37 plant species including identification of 6788 potato (*Solanum tuberosum*) lncRNAs [11]. However, to date, investigation of the pervasive transcription in intergenic regions in potato and identification of lincRNAs have not yet been done on a genome-wide scale.

Potato is an important staple crop ranking fourth in global production after maize, rice and wheat. It can be severely affected by soft rot *Enterobacteriaceae* (SRE) species, in particular, *Pectobacterium carotovorum* subsp *brasiliense* (*Pcb1692*), an emerging member of the SRE, which is the most important causal agent of potato blackleg and soft rot globally including South Africa. Consequently, this pathogen poses a major threat to the potato industry in terms of yield, tuber quality and tuber seed exports [27]. Pathogen-responsive lincRNAs have been implicated in defence responses against *Fusarium oxysporum* infection in Arabidopsis [9], and powdery mildew infection responses in wheat [8]. Given the importance, albeit not well characterized, of lincRNAs in plant response to these pathogens, it would be interesting to unravel the repertoire of lincRNAs in potato and identify those responsive to this important emerging soft rot bacterium.

We identified 1113 potato candidate lincRNAs present in two potato cultivars that are susceptible (*S. tuberosum* cv. Valor) and tolerant (*S. tuberosum* cv. BP1) to *Pcb1692*. Using potato time-course RNA-seq data following infection with *Pcb1692*, we identified 559 potato lincRNA candidates that showed significant differential expression in the stems of the resistant and susceptible cultivars, compared to the mock-inoculated samples. Of these, six were validated using RT-qPCR. Importantly, expression of 17 lincRNAs was highly correlated with potato defence-related genes. Thus, our results suggest that lincRNAs are involved in potato defence mechanisms.

## Methods

### Plant material and growth conditions

Seed tubers of two potato cultivars, susceptible (*Solanum tuberosum* cv. Valor) and tolerant (*S. tuberosum* cv. BP1) to *Pectobacterium carotovorum* subsp *brasiliense* strain 1692 (*Pcb1692*) infection were grown in the greenhouse under standard conditions (22 to 26 °C, 16 h light/ 8 h dark photoperiod and 70 % relative humidity). Stem inoculations were done as previously described in Kubheka et al. [28], except that we used wild-type *Pcb1692* for the inoculations and inoculated plants were assessed and sampled at 0, 6, 12, 24, and 72 h post inoculation (hpi) in triplicates (three plants were pooled together for each biological replicate).

### Total RNA preparation

Total RNA was extracted from potato stems using the QIAGEN RNeasy plant mini kit (Qiagen) including DNAse treatment (Qiagen). RNA was quantified using the NanoDrop (Thermo Scientific, Sugarland, TX, USA) and the quality and integrity checked using Agilent 2100 BioAnalyzer system (Agilent, Santa Clara, CA, USA).

### Whole transcriptome library construction and sequencing

The construction of whole transcriptome libraries and sequencing were carried out at the Beijing Genomics Institute (BGI-Shenzhen, China). For the preparation of strand-specific libraries, total RNA was pooled from five time-points (0, 6, 12, 24, 72 hpi) for BP1 and Valor. Whole transcriptome libraries were constructed using the TruSeq Stranded RNA Sample Prep Kit v2 (Illumina, San Diego, CA), according to the manufacturer’s instructions. For the time-course experiment, standard (normal) transcriptome libraries were constructed using RNA samples from individual biological replicates (*n* = 3) from each time-point using the TruSeq RNA sample Prep Kit v2 (Illumina, San Diego, CA) following manufacturer’s instructions. The libraries were quality checked and quantified using Agilent BioAnalyzer 2100 system and qPCR. Finally, the libraries were sequenced on the Illumina HiSeq 2000 system generating 90 bp paired-end reads. The data have been deposited in NCBI’s Gene Expression Omnibus (GEO) and are accessible through the GEO accession number, GSE74871.

### Assembly of RNA transcripts

Strand-specific sequencing reads for each cultivar were quality checked using FASTQC (http://www.bioinformatics.bbsrc.ac.uk/projects/fastqc) and mapped to the potato reference genome (Genome assembly: PGSC_DM_v4.03; http://solanaceae.plantbiology.msu.edu/pgsc_download.shtml) using TopHat2 (version 2.0.13) [−−library-type fr-firststrand –G] [29]. For the alignments, the minimum (−i) and maximum (−l) intron sizes were obtained at http://solanaceae.plantbiology.msu.edu/pgsc_download.shtml, and set at 10 bp and 15,000 bp, respectively. Transcript assembly was performed using Cufflinks (version 2.2.1) [-g -u --library-type fr-firststrand] [30].

### Bioinformatics identification of lincRNAs

The assembled potato transcripts were compared with annotated potato protein sequences (http://potato.plantbiology.msu.edu/data/PGSC_DM_V403_representative_genes.gff.zip) using IntersectBed (v2.22.1) [31]. All assembled transcripts overlapping with potato coding sequences and less than 200 bp from protein coding regions were removed. For size selection, java scripts were used to filter out all transcripts less than 200 nucleotides in length. For the sequencing depth filter, HTSeq-count [python -m HTSeq.scripts.count -f bam -s reverse] [32] was used and only transcripts with at least two reads were considered. Following sequencing depth filter, IntersectBed (v2.22.1) [31] and stringent Blastn (Evalue: 1.0E-100) was used to extract novel transcripts present in both BP1 and Valor. Since lncRNA transcripts are generally known not to have any coding capacity, all the transcripts common to BP1 and Valor were tested for protein-coding potential using the Coding Potential Calculator (CPC) [33]. Following the coding potential filter, only transcripts with a negative CPC score were retained as potential novel lincRNA candidates.

### Distribution of lincRNAs and protein-coding genes in the potato genome

A circular representation of the distribution of lincRNAs and mRNAs was constructed using Circos [34] for comparative visualizations among the 12 chromosomes.

### Classification of lincRNAs

Potato lincRNAs were classified into three categories based on their genomic location and distance from protein-coding genes nearest to each lincRNA transcript using IntersectBed (v2.22.1) [31] and java scripts. The lincRNAs were grouped into: 1) intergenic-lncRNA, without any overlaps with protein-coding genes on both strands and at least 1 kb away from the nearest CDS 2) adjacent-lncRNA, which are in close proximity to protein coding genes but without any overlaps and 3) antisense-lncRNA, which partially overlap with genes on the opposite strand.

### Differential expression analysis of lincRNAs between the tolerant and susceptible potato cultivars

Time course RNA-seq data from stems of BP1 and Valor was used to identify lincRNAs responsive to *P. carotovorum* subsp. *brasiliense* infection. Briefly, to identify differentially expressed lincRNAs between Valor and BP1, RNA-seq reads were quality checked using FASTQC and mapped to the potato reference genome using TopHat2 [29]. HTSeq-count was used to make read counts mapped to lincRNA transcripts and DeSeq2 [35] was used to determine the differential expression with a false discovery rate threshold of 10 %.

### Quantitative reverse transcription PCR (RT-qPCR)

For RT-qPCR, first-strand cDNA synthesis was done from total RNA using Superscript III First-Strand cDNA Synthesis SuperMix kit (Invitrogen, USA) following manufacturer’s instructions. Quantitative real-time PCR using Applied Biosystems SYBR Green Master Mix was performed in the QuantStudio 12 K Flex Real-Time PCR system (Life Technologies, Carlsbad, CA, USA). For RT-qPCR, 2 μl of sample was added to 8 μl of Applied Biosystems SYBR Green Master Mix and primers at a concentration of 0.4 μM. The cycling conditions were as follows: an initial denaturation at 50 °C for 5 min and 95 °C for 2 min followed by 45 cycles of 95 °C for 15 s and 60 °C for 1 min. Each sample was run in triplicate and two biological replicates were employed. The samples were normalized to 18S rRNA and elongation factor 1-α (ef1α) as the reference genes [36] and the mock treated samples used as calibrators. The comparative CT (ΔΔ^ct^) method was used to measure relative expression [37]. Primers used were designed online using Primer3Plus (http://primer3plus.com/cgi-bin/dev/primer3plus.cgi) and are listed in Additional file 1: Table S5.

### LincRNA-mRNA coexpression analysis

To investigate correlations of expression between lincRNA and differentially expressed mRNA transcripts under the same conditions, an all-against-all hierarchical clustering analysis was performed based on log2 fold changes using Cluster 3.0 software [38]. Briefly, LincRNA and mRNA datasets were filtered so that only transcripts with an expression of at least 2-fold at any of the 5 time-points tested were considered. Clustering was performed using the Spearman Rank Correlation similarity metric (r_*rho*_ > |0.8|) and the complete linkage clustering method. Visualization was done using TreeView program [39]. In order to predict potential lincRNA functions, mRNA transcripts grouped together with lincRNAs in various clusters were used to perform Gene Ontology (GO) analysis based on the Panther Classification System (version 10.0) web server [40]. Corresponding orthologs in Arabidopsis of the differentially expressed potato mRNA genes were used for the GO enrichment analysis, based on BLASTp (e-value: 1.0E-05), implemented in ProteinOrtho program [41]. Lastly, pairwise Spearman correlation coefficient was calculated by cor.test() in R, between CDS genes and lincRNAs grouped within each cluster associated with response to stimulus GO biological process terms. To assign putative functional annotations to the lincRNAs, GO terms of CDS genes significantly correlated with lincRNAs were mapped to the lincRNAs.

### RT-PCR validation of lincRNA transcripts

First-strand cDNA was synthesized as described above for RT-qPCR and the PCR was performed on Bio-RAD T100TM Thermal Cycler conventional PCR (Bio-RAD, USA). The lincRNA primers were designed online using Primer3plus (Additional file 2: Table S6). PCR was performed in a 25 μl reaction mix containing 1 μl of template cDNA (~40 ng), Taq DNA Polymerase, 10x Taq Buffer (New England Biolabs, UK), 2.5 mM dNTPs each and 0. 5 μM of forward and reverse primer each. Thermal cycling conditions were: 95 °C for 2 min; 30 cycles of 95 °C for 30 s, 57 °C for 30 s, 72 °C for 60 s, and the final extension at 72 °C for 5 min. The PCR products were analysed on 1.5 % agarose gel including 1 kb DNA molecular weight ladder 470 (NEB, UK). To check for genomic DNA contamination, a non reverse-transcriptase control was included.

### Prediction of lincRNA and miRNA interactions

Potato lincRNAs targeted by miRNAs were predicted using the psRNATarget [42] server by using default parameters.

## Results

### Genome-wide identification of lincRNAs in potato

**Fig. 1:**
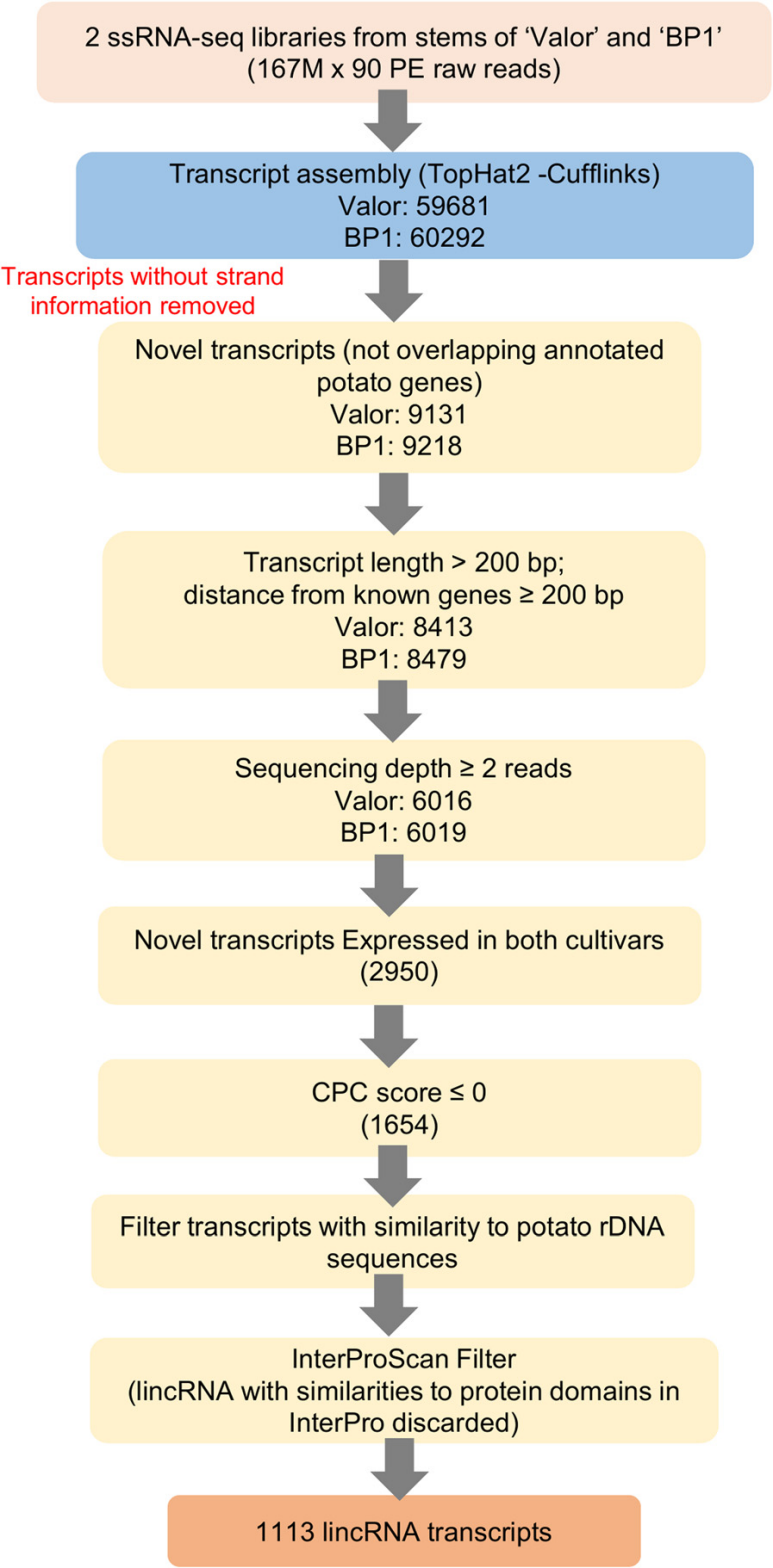
Schematic diagram of the bioinformatics approach used for identification of potato lincRNAs

In order to identify long intergenic noncoding RNA (lincRNAs) related to potato defence networks, we employed a computational approach using strand-specific RNA-seq (ssRNA-seq) data derived from stems of *Solanum tuberosum* cultivars Valor and BP1 (Fig. 1). Samples of potato stems of each cultivar were harvested from six time-points post inoculation with *Pcb1692*, RNA isolated and pooled together. Sequencing was conducted on representative RNA pools of the susceptible and tolerant cultivars. The ssRNA-seq generated approximately 36 million (33 million uniquely mapped) and 38 million (35.3 million uniquely mapped) paired-end reads in *S. tuberosum* cvs Valor and BP1, respectively. From these data, a computational strategy was used that enabled the identification of lincRNAs after read mapping and transcript abundance assembly using Tophat2 (v2.0.13) and Cufflinks (v2.2.1), respectively [29, 30]. As an initial step, all transcript loci, from the potato genome annotation without strand information were removed prior to performing read alignments and transcript assembly. Subsequently, 59,681 and 60,292 transcripts were reconstructed for *S. tuberosum* cvs Valor and BP1, respectively. The majority of the assembled transcripts (84.7 %) represented annotated genes and allelic isoforms in the potato reference genome assembly (PGSC_DM_v4.03) for both *S. tuberosum* cvs Valor and BP1.

### Identification of novel transcriptionally active regions

**Fig. 2:**
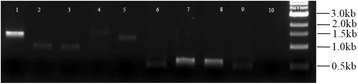
RT-PCR validation of nine lincRNA transcripts. Agarose gel electrophoresis of the PCR amplicon fragments representing each lincRNA. Lane 1. LincRNA9, Lane 2. LincRNA10, Lane 3. LincRNA12, Lane 4. LincRNA13, Lane 5. LincRNA20, Lane 6. LincRNA178, Lane 7. LincRNA1405, Lane 8. LincRNA24, Lane 9. LincRNA1102, Lane 10. No reverse transcriptase control, Lane 11. 1 kb DNA ladder

To identify transcripts representing novel transcriptionally active regions (TARs) from the Cufflinks assembled transcripts, we first eliminated all the transcripts that overlapped with annotated potato features on the same strand. Our main focus in this study was particularly to identify novel noncoding RNA transcription; thus, only transcripts at a distance of more than 200 nucleotides from known genes on the same strand were considered, with lengths above 200 bp. Furthermore, to eliminate the possibility of genomic DNA contamination, only transcripts with a sequencing depth of at least two reads per transcript were retained (Fig. 1). Additionally, since we were interested in measuring and comparing the variation in transcript abundances of the TARs between *S. tuberosum* cvs Valor and BP1 using time-course RNA-seq data in our downstream analysis, we started by first determining novel transcripts that were common between both the cultivars based on strand-specific RNA-seq data. Using the IntersectBed tool (v2.22.1) [31] and Blastn (Evalue: 1.0E-100) we identified 2950 novel transcripts that were present in the two cultivars. To determine a set of long intergenic noncoding RNA transcripts (lincRNAs) that is novel, the coding capability of these transcripts was then assessed using the Coding Potential Calculator (CPC) [33]. CPC evaluates the protein-coding potential of transcripts based on prediction and assessment of potential open reading frames (ORFs) features and BLASTX (E-value cut-off 1.0E-10) homology searches against the non-redundant Uniprot Reference Clusters (UniRef90) protein database. Based on the extracted feature information, CPC algorithm, uses a score to classify transcripts into either protein-coding or noncoding. In this regard, all the transcripts showing evidence for protein-coding (CPC score > 0) were eliminated. Consequently, we obtained 1654 lincRNAs expressed in both potato cultivars with CPC scores less than zero. The 1654 lincRNAs obtained were further filtered to remove any lincRNAs with similarity to potato ribosomal DNA sequences (obtained from EnsemblPlants SolTub_3.0 Assembly (Blastn: E-value 1.0E-2)) resulting in 1649 lincRNA candidates. Of these, 1177 were high-confidence novel lincRNAs (CPC score < −1) and 472 were weak-novel lincRNA based on the CPC scores (Additional file 3: Table S1). However, because CPC uses a stringent Blastx cutoff (E-value: 1.0E-10), and only performs similarity analysis against the UniRef90 protein database, it is possible that some mRNA transcripts with relatively weak protein signatures could be falsely classified as potential lincRNA transcripts. Thus, we further screened the 1649 lincRNA candidates against InterPro [43], using InterProScan5 [44]. LincRNA sequences with similarities to protein families and domains from any of the databases within the InterPro consortium were considered as protein-coding and eliminated. Finally, following the additional filters, a list of 1113 transcripts was regarded as novel lincRNA potato transcripts expressed in stems of *S. tuberosum* cvs Valor and BP1 (Additional file 3: Table S1). Semi-quantitative reverse transcription (RT)-PCR confirmed nine of the RNA-seq identified lincRNAs, thus validating the assembly quality and identification pipeline (Fig. 2).

### Characterization and classification of potato lincRNAs

Using basic features of the identified lincRNAs in a genomic context, we found that the lincRNAs ranged from 200 to 17,256 bp in size and were transcribed from all the 12 potato chromosomes (Potato Genome Assembly: PGSC_DM_v4.03) (Additional file 4: Figure S1A). The highest and least numbers of lincRNAs were transcribed from chromosome one (183 lincRNAs) and chromosome 12 (nine lincRNAs), respectively. As with mRNA transcripts, lincRNAs appeared to be distributed uniformly across all chromosomes, with the exception of chromosome 12, were lincRNAs were only concentrated within the region up to 5 Mbp (Additional file 4: Figure S1A). In addition, based on length distribution, lincRNAs can be divided into three groups, namely, short-length, medium-length and long-length lincRNAs [4]. Thus, the majority of potato lincRNAs (71 %) are short-length lincRNA (200–1000 bp), 26 % are medium-length lincRNA (1–5 kb) and only 3 % are long-length lincRNA (>5 kb). In contrast, most of the protein-coding transcripts, 54 % comprise medium transcripts (Additional file 4: Figure S1B). Comparing the number of exons between annotated potato genes and lincRNAs showed that on average, lincRNAs possess fewer exons (Table 1). Furthermore, we assessed the repeat content (including presence of transposons) of potato lincRNAs using RepeatMasker (http://www.repeatmasker.org) and the TIGR *Solanum* Repeat Database v3.2 (plantrepeats.plantbiology.msu.edu/downloads.html). Almost half of the lincRNAs (42.3 %) contain repetitive sequences.

**Table 1:** Exon numbers of lincRNA and protein-coding genes in *Solanum tubersoum* cvs Valor and BP1

	0	1	2	3	4	5	6	7	8	9	≥10	Total
Valor	5	791	145	93	32	11	9	4	6	6	10	1113
BP1	17	779	158	74	33	11	14	5	9	3	10	1113
^a^mRNA	16883	6937	3561	2753	1960	1599	1144	937	709	621	1924	39 028

^a^mRNA exons obtained from the potato genome assembly (PGSC_DM_v4.03)

**Fig. 3:**
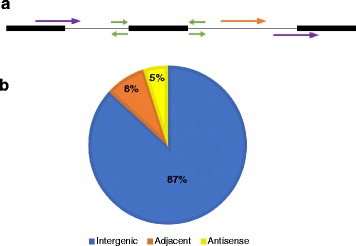
Classification of potato lincRNAs relative to protein-coding transcripts. **a** A schematic diagram showing the location of lincRNAs in relation to adjacent protein-coding genes (*black rectangles*). *Purple arrows* represent antisense-lncRNAs which overlap annotated genes on the opposite strand; *Green arrows* show adjacent-lncRNAs which are positioned in close proximity to annotated genes; and the *Orange arrow* represents long intergenic noncoding RNAs (lincRNAs). **b** Percentage and distribution of lincRNAs in three classes

Even though it is still not yet clear how classification of lncRNAs based on their proximity to coding genes reflects biological function, knowing where lncRNAs are located in the genome and their expression profiles provides useful insights into their biological significance and primary mechanisms of action [45]. Thus, we classified the identified lincRNAs into three types, based on their genomic location and proximity with respect to their closest protein-coding genes, namely: intergenic (distance > 1 kb; without any overlaps with CDSs on both strands), adjacent (distance < 1 kb) and antisense-lncRNAs (those partially overlapping protein-coding genes on the opposite strand) (Fig. 3a). Most of the lincRNAs (87 %) are located at least 1 kb away from annotated potato gene models on either strand, 8 % of lincRNA are adjacent-lncRNA, located in close proximity to protein-coding genes, and only a small proportion (5 %) constituted antisense-lncRNAs (Fig. 3b and Additional file 3: Table S1). The percentage difference observed between lincRNAs at distance > 1 kb from CDS regions and antisense-lncRNA is consistent with previous observations made in maize [10] and tomato [25].

Furthermore, the identified lincRNA sequences were compared with lncRNA sequences from tomato [25], Populus [22], and Arabidopsis [21, 46, 47] to determine the set of potato lincRNAs with similarity to these plant species (BLASTn e-value < 1.0E-10). As expected, the lincRNAs displayed poor conservation. Only 13 % lincRNAs showed multiple homologous regions (>80 % identity and alignment length > 100 bp) with 231 lncRNAs from tomato and two from Arabidopsis (Additional file 5: Table S2). Thus, unlike most mRNAs which are highly conserved across organisms, lincRNAs tend to evolve rapidly resulting in poor conservation [48]. Lastly, to check the novelty of our set of lincRNAs, we checked for overlaps against potato lncRNAs reported by Gallart et al. [11]. Comparisons using IntersectBed tool (v2.22.1), showed that only nine lincRNAs out of the 1113 lincRNAs from our set overlapped with and were similar to nine previously reported potato lncRNAs (Additional file 6: Figure S2).

### Quantitative analysis of potato lncRNAs responsive to *Pectobacterium carotovorum* subspecies *brasiliense* infection

**Fig. 4:**
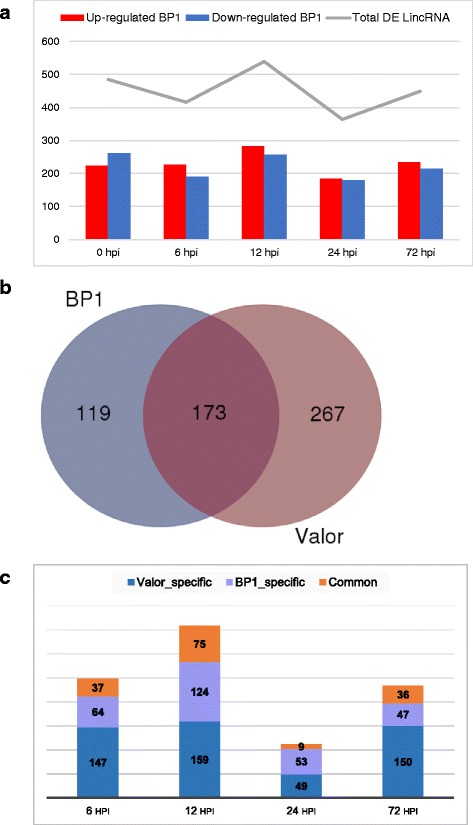
LincRNAs significantly expressed over time between Valor and BP1 following infection with Pcb1692. **a** Pairwise comparisons between S. tuberosum cv Valor and S. tuberosum cv BP1 at each time-point. *Red* represent significantly upregulated and *blue* represent significantly downregulated. **b** Comparison of DE lincRNAs specific to each cultivar in relation to the mock-inoculated samples (0 hpi). **c** Numbers of DE lincRNAs common or specific to each cultivar at individual time-points in relation to mock inoculated samples

From our previous work [28], we showed that *S. tuberosum* cv. Valor is highly susceptible to *Pcb1692* infection showing typical blackleg symptoms upon infection. On the other hand, *S. tuberosum* cv. BP1 was shown to be tolerant to *Pcb1692*. Furthermore, gene expression analysis performed in our lab between Valor and BP1 revealed differentially expressed protein-coding genes involved in potato defence responses to *Pcb1692* infection (unpublished data). However, it remains to be investigated whether lincRNA expression is activated in response to *Pcb1692* infection in potato. Thus, we hypothesized that lincRNAs could be involved in potato defence mechanisms and therefore differentially expressed in the tolerant compared to susceptible cultivar. Consequently, in the present work, we sought to determine novel lincRNA transcripts that were differentially expressed between the two cultivars and could thus be implicated in potato defences against the necrotrophic plant pathogen, *Pcb1692*. Our bioinformatics analysis showed that a total of 1113 lincRNAs were expressed in both cultivars. Thus, to identify defence-related lincRNAs, the expression levels of these commonly expressed 1113 lincRNAs were compared between the two cultivars, *S. tuberosum* cvs Valor and BP1. To do this, each cultivar was inoculated with *Pcb1692* (1 × 10^9^ cfu.ml^−1^) and samples obtained at five different time points (0 (Buffer inoculated control), 6, 12, 24, 72 h post inoculation; hpi). RNA was isolated from each time point and three biological replications per time point were prepared and sequenced independently. Using the resulting time-course RNA-seq data, the differential expression patterns of the 1113 lincRNAs was evaluated. In total, 485, 416, 539, 364, and 449, lincRNAs were differentially expressed (DE) at 0, 6, 12, 24, and 72 hpi, respectively, between *S. tuberosum* cvs *Valor* and *BP1*, at 10 % false discovery rate (FDR) (Additional file 7: Table S3). Compared with *S. tuberosum* cv *Valor*, an average of 51 % lincRNAs were upregulated in *S. tuberosum* cv *BP1*, throughout the time-course. Numbers of up-regulated lincRNAs in the tolerant cultivar were slightly higher at 6, 12 and 72 hpi, with the most up-regulated lincRNAs observed at 6 hpi (54 %) (Fig 4a). Furthermore, in order to determine the expression profiles of these differentially expressed lincRNAs showing cultivar-specific differences, DE lincRNAs at each time-point (post inoculation) were compared to mock-inoculated samples (0 hpi) in each cultivar. In total, 173 DE lincRNAs were present in both cultivars and 267 and 119 were only significantly expressed in *S. tuberosum* cv *Valor* and *S. tuberosum* cv *BP1*, respectively (Fig. 4b and c).

**Fig. 5:**
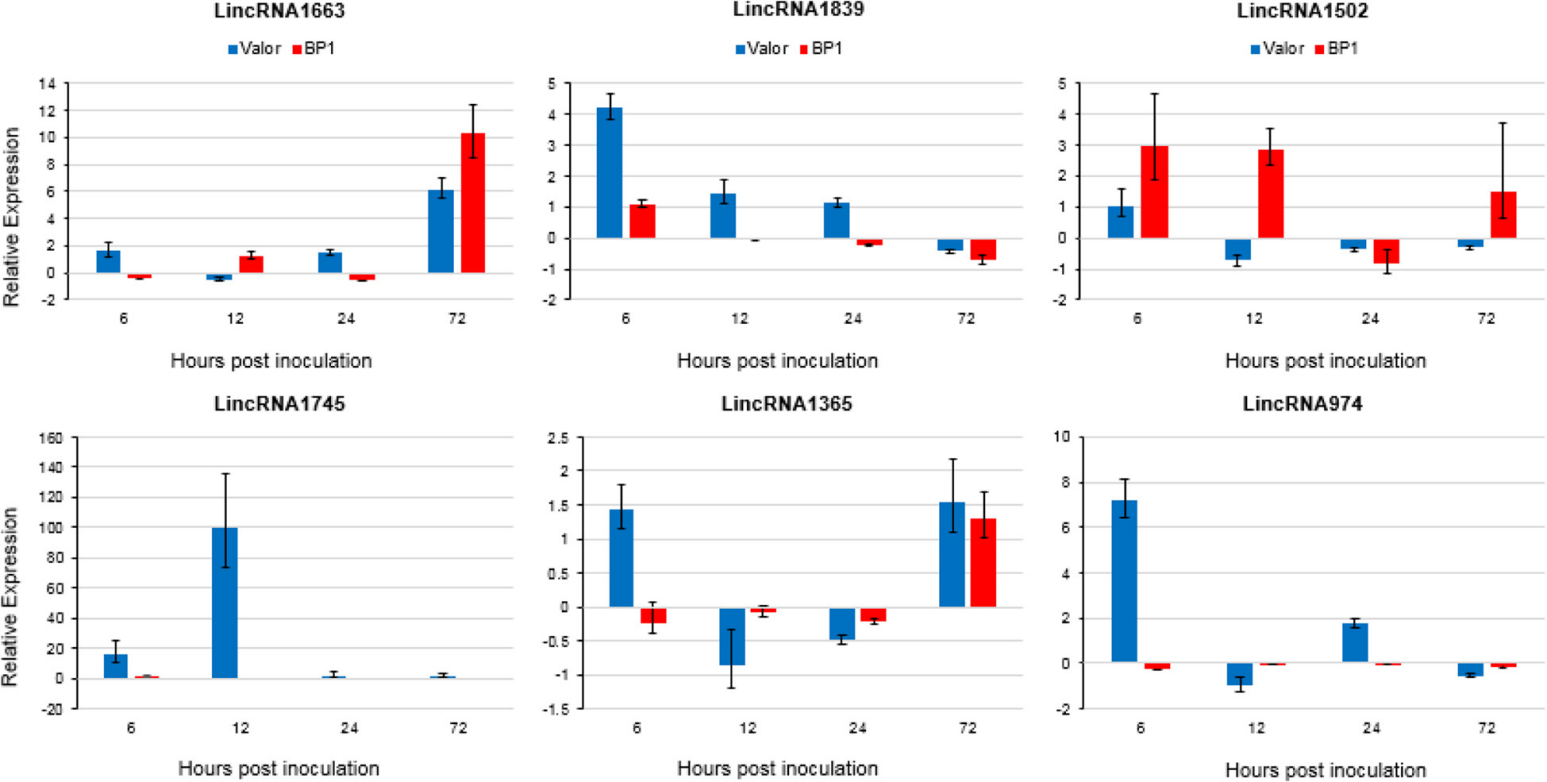
RT-qPCR validation of time-course RNA-seq data using six selected lincRNAs differentially expressed over time. 18S rRNA and elongation factor 1-α (ef1α) were used as the reference genes. The relative expression levels of lincRNAs at each time point were calculated relative to calibrator (control sample; 0 hpi). Error bars represent the range of relative expression (fold change) calculated by 2^-(ΔΔCt±SD)^. Two biological replicates were used in triplicate

To confirm RNA-seq expression patterns and determine whether the differentially expressed lincRNAs are involved in potato defence responses, six of these lincRNAs were arbitrarily selected representing lincRNAs that were up or down regulated at one or more time-points and validated experimentally using reverse-transcription quantitative PCR (RT-qPCR) (Fig. 5). The RT-qPCR results were in concordance with the RNA-seq data, thus, implicating the DE lincRNAs in potato defence responses.

### LincRNA/mRNA genes expression correlation

In order to understand the possible biological roles of the differentially expressed (DE) lincRNAs in relation to potato defence responses, we investigated all-against-all coexpression patterns between lincRNA transcripts and DE mRNA genes within the time-course using hierarchical clustering. In total, 179 lincRNAs were highly correlated with 3573 mRNA genes (Spearman rank correlation (r_*rho*_) > |0.8| and were clustered into 62 different groups. Interestingly, Gene Ontology (GO) enrichment analysis using the Panther Classification System web server [40], showed that 32 clusters contained CDS genes enriched in “response to stimulus”, including secondary GO terms such as “defence response to bacterium”, “response to stress” and “response to endogenous stimulus”. Therefore, to highlight potential lincRNA functions and/ or interactions with CDS genes involved in potato defence mechanisms, we further performed pairwise correlations between CDS genes and lincRNAs within each cluster associated with response to stimulus GO terms. Overall, 17 lincRNAs exhibited extremely high positive correlation (*r*_*rho*_ ≥ 0.9) with 12 potato defence-related CDS genes (Table 2). These results suggest that these highly correlated lincRNAs could be involved in potato defence responses against *Pcb1692* infection.

**Table 2:** LincRNA transcripts highly coexpressed with defence-related CDS genes

LincRNA ID	LincRNA class	*r*_*rho*_^a^	Potato gene ID	Arabidopsis ortholog	Gene description	Gene Ontology classification (Biological process)	
LincRNA739	Intergenic	1	PGSC0003DMG400014801	AT4G17760	rad1-like	response to stimulus (GO:0050896)	response to stress (GO:0006950)
LincRNA1304	Intergenic	1	PGSC0003DMG400020345	AT2G38080	Laccase-4	response to stimulus (GO:0050896)	response to toxic substance (GO:0009636)
LincRNA127	Intergenic	1	PGSC0003DMG400011631	AT3G46230	17.4 kDa class I heat shock protein (HSP17.4A)	response to stimulus (GO:0050896)	response to stress (GO:0006950)
LincRNA803	Intergenic	1		AT1G53540	17.6 kDa class I heat shock protein 3 (HSP17.6C)	response to stimulus (GO:0050896)	response to stress (GO:0006950)
LincRNA127	Intergenic	1	PGSC0003DMG400000996	AT3G47570	Probable LRR receptor-like serine/threonine-protein kinase	response to stimulus (GO:0050896)	defence response to bacterium (GO:0042742)
				AT3G47580	Leucine-rich repeat protein kinase family protein		
				AT3G47090	Leucine-rich repeat protein kinase-like protein		
LincRNA1464	Intergenic	1	PGSC0003DMG400012994	AT5G21950	Hydrolase, alpha/beta fold family protein	response to stimulus (GO:0050896)	response to toxic substance (GO:0009636)
				AT4G33180			
LincRNA907	Intergenic	1	PGSC0003DMG400000757	AT2G23620	Methylesterase 1	response to stimulus (GO:0050896)	response to toxic substance (GO:0009636)
LincRNA1118	Intergenic	0.9					
LincRNA258	Intergenic	0.9					
LincRNA632	Intergenic	1	PGSC0003DMG400025635	AT3G45920	Protein kinase family protein		defence response to bacterium (GO:0042742)
			PGSC0003DMG400004885	AT2G24370	Adenine nucleotide alpha hydrolase domain-containing protein kinase		defence response to bacterium (GO:0042742)
LincRNA758	Intergenic	1					
LincRNA749	Intergenic	1					
				AT4G09570	Calcium-dependent protein kinase 4		response to endogenous stimulus (GO:0009719)
			PGSC0003DMG400026077	AT1G35670	Calcium-dependent protein kinase 11		
LincRNA758	Intergenic	1					
LincRNA749	Intergenic	1					
			PGSC0003DMG400013679	AT1G30270	CBL-interacting serine/threonine-protein kinase 23	response to stimulus (GO:0050896)	
LincRNA1112	Intergenic	1					
LincRNA908	Intergenic	0.9					
LincRNA1712	Intergenic	0.9					
			PGSC0003DMG400030755	AT1G77110	Probable auxin efflux carrier component 6	response to stimulus (GO:0050896)	response to endogenous stimulus (GO:0009719)
LincRNA178	Intergenic	0.9					
LincRNA1583	Intergenic	1					
			PGSC0003DMG400021008	AT4G20940	Probable LRR receptor-like serine/threonine-protein kinase	response to stimulus (GO:0050896)	defense response to bacterium (GO:0042742)
LincRNA379	Intergenic	0.9					

^a^Spearman correlation coefficient

### Prediction of interactions between lincRNAs and miRNAs

Long noncoding RNAs can be involved in diverse cellular molecular functions depending on their mode of action [4]. Because lincRNAs are functional RNA molecules, they can be targeted and regulated by miRNAs post transcriptionally, triggering degradation of the targeted lincRNAs. To investigate whether the identified potato lincRNAs are targeted by miRNAs, we analyzed the 1113 lincRNAs using psRNATarget [42]. A total of 57 lincRNAs were predicted to be targeted by 98 potato miRNAs (Additional file 8: Table S4). Of these lincRNAs, four were targeted by six miRNAs implicated in plant immune defences [49] (Additional file 8: Table S4). Interestingly, none of these four lincRNAs were differentially expressed in the time-course following inoculation with *Pcb1692* possibly reflecting their miRNA mediated cleavage and degradation. RT-qPCR analysis confirmed expression of these defence-related miRNAs under the same experimental conditions, adding credence to their possible interaction or regulation of their target lincRNAs (Additional file 9: Figure S3). An additional 10 lincRNAs were targets of various members of stu-miR5303 family which is part of nine miRNA families unique to solanaceous plants [50] (Additional file 8: Table S4). Previously identified potential targets of stu-miRNA5303 family include proteins responsive to abiotic stress, metabolic enzymes and proteins of unknown function [50]. The large numbers of stu-miRNA5303 members implies its biological importance. Consequently, it is plausible to assume that their target lincRNA transcripts play important biological roles in potato.

## Discussion

The regulatory roles of lincRNAs are increasingly being unraveled in plants, as indicated by the number of various reports on the identification of lncRNAs in plant species including maize, millet, rice, *Populus* and Arabidopsis [7, 10, 12, 21–23, 26]. However, most of these reports have focused on lncRNAs involved in plant development, reproduction and abiotic stress responses [3, 10, 12, 13, 51]. In contrast, reports about lincRNAs involved in defence regulatory mechanisms against pathogens are just beginning to emerge [8, 9]. In potato, previous studies on noncoding RNA have predominantly focused on miRNA identification and functional analysis [49, 52–54], but no data have been reported for lincRNAs, especially in association with potato defence responses. In this study, we conducted a genome-wide analysis of potato lincRNAs, by integrating strand-specific RNA sequencing with time-course RNA-seq data. We identified novel candidate lincRNAs potentially associated with potato defence response mechanisms during challenge by *Pcb1692*. Hence, this present work provides an important resource of potato lincRNAs that can be useful to other researchers.

To facilitate the identification of lincRNAs, a strand-specific RNA-seq (ssRNA-seq) approach was employed which made it possible to determine the strand from which the lincRNAs were produced. This is in contrast to some previous lincRNA identification reports in plants, which had the limitation of RNA-seq data lacking strand information [10, 22]. Knowledge of the strand information of lncRNAs is important in localizing their genome context and position since lincRNAs are transcribed from intergenic regions with some being adjacent or antisense to protein-coding regions [55]. Thus, strand-specific RNA-seq allowed us to classify the identified lincRNAs into three categories, based on their proximity to protein-coding genes (Fig. 3b). Classification of lincRNAs based on their genomic location can be a useful preliminary step in determining potential functional roles of lincRNAs [4]. In addition, our present work revealed that, like protein-coding genes, lincRNAs are distributed throughout the potato genome (Additional file 4: Figure S1A). Thus, the pervasive expression of lincRNAs in the entire 12 potato chromosomes suggests that they are common RNA molecules representing a functional component of the potato genome.

Additionally, by aligning the ssRNA-seq reads, using Tophat2, a splice-aware aligner and performing transcript reconstruction using the software tool Cufflinks [30], a parsimonious representation of exon boundaries for the lincRNAs was obtained. As a result, the structure of lincRNA transcripts was resolved. Furthermore, since consideration was only given to lincRNA candidates conserved in *S. tuberosum* cvs. Valor and BP1, the identified 1113 lincRNAs constitute a reliable list of lincRNAs from potato stems, extending the current understanding of the potato transcriptome landscape.

In general, functional characterization of lincRNAs in plants is still in its infancy. Moreover, little is known about regulatory functions of lincRNAs in biotic stress responses in plants. Currently, the function of lncRNAs cannot be inferred directly from primary sequence or structure as is the case with miRNAs and protein-coding mRNA [56]. However, key insights into biological roles of lncRNAs can be derived from the conditions in which they are expressed [45]. In the present study, we identified 559 differentially expressed (DE) lincRNAs at different time-points (up to 72 hpi) in *S. tubersom* cvs. Valor and BP1, compared to mock-inoculated samples (Fig 4b). Meanwhile, the responsiveness of six DE lincRNAs to *Pcb1692* infection was also confirmed using RT-qPCR, further alluding to the potential functional activity of these lincRNAs.

From a systems biology perspective, the guilt-by-association principle has been applied successfully for the functional characterization of various genes in humans and other mammals assuming functional relationships between co-expressed genes [57, 58]. Thus, in the present study, a hierarchical clustering strategy was employed in order to infer potential functional roles of DE lincRNAs in the time-course, and enriched functions of CDS genes within individual clusters were identified. Only clusters with genes enriched for biological process GO terms under the “response to stimulus” category were considered. Generally, coexpression analysis is used to predict biological processes and infer novel members (genes, ncRNA transcripts etc.) of known processes and/or pathways [59]. In addition, identifying lincRNAs associated with the “response to stimulus” category was particularly relevant in this study due to the fact that most defence-related genes are often overrepresented within this category. Therefore, GO terms of CDS genes that showed significantly high pairwise coexpression with lincRNAs (r_*rho*_ > 0.9) within the clusters, were mapped to lincRNAs (Table 2). Based on this analysis, 17 lincRNAs were co-expressed with genes associated with defence-related GO terms such as response to stimulus, defence response to bacterium, response to endogenous stimulus, response to stress and response to toxic substance (Table 2). Most of these CDS genes belong to the Leucine-rich repeat protein kinase family, which mainly function as pattern recognition receptors in plant innate immune responses. Thus, our results implicate these co-expressed lincRNAs in defense responses against *Pcb1692* in the tolerant cultivar, making them key candidates for future experimental validations.

Interestingly, these 17 lincRNAs are located more than 100 kb from their correlated CDS genes, and some of them are interchromosomal with regards to CDS genes they are co-expressed with. Thus, these lincRNAs are possibly *trans*-acting, functioning as transcriptional regulators that interact with genes at distal locations across multiple chromosomes. However, there is a paucity of information regarding molecular mechanisms employed by lincRNAs in regulating their distal gene targets. Nonetheless, we anticipate that these mechanisms will become more apparent in the near future.

## Conclusions

This study focused on the genome-wide discovery of lincRNAs using strand-specific RNA-seq and resulted in the first catalogue of potato lincRNAs, comprising 1113 transcripts, including 1104 novel lincRNA candidates, derived from stem tissue. In addition, we identified 559 lincRNAs that were responsive to *P. carotovorum* subsp. *brasiliense* infection in *S. tuberosum* cvs *Valor* and *BP1*. Importantly, 17 differentially expressed lincRNAs were highly associated with defence-related CDS genes, thus representing key candidates for future functional studies.

## Abbreviations

CDS, protein-coding genes; LincRNA, long intergenic noncoding RNA; lncRNAs, long noncoding RNAs; miRNAs, microRNAs; NATs, Natural antisense transcripts; ncRNAs, noncoding RNAs; *Pcb1692*, *Pectobacterium carotovorum* subsp. *brasiliense; r*_*rho*_, Spearman Rank correlation coefficient; RT-PCR, reverse transcription PCR; RT-qPCR, quantitative reverse transcription PCR; siRNAs, small interfering RNAs; snoRNAs, small nucleolar RNAs; snRNAs, small nuclear RNA; SRE, soft rot *Enterobacteriaceae;* ssRNA-seq, strand-specific RNA sequencing

## Additional files

Additional file 1: Table S5. List of RT-qPCR primers used in this study. (XLS 26 kb) Additional file 2: Table S6. List of RT-PCR primers used in this study. (XLSX 8 kb) Additional file 3: Table S1. List of the identified 1113 lincRNA candidates. (XLS 166 kb) Additional file 4: Figure S1. A) Comparison of the genomic distribution of lincRNAs and protein-coding genes across the 12 potato chromosomes. The outer grey track represents the 12 potato chromosomes, with a scale (Mb) showing the length of each chromosome. The red histograms (second track with an outer orientation) and blue histograms (third track with inner orientation) represent the abundance and distribution of mRNA and lincRNAs, respectively, throughout the potato genome. The bin size (histogram width) = 5 Mbp). B) Comparison of LincRNA lengths to protein-coding mRNA transcripts in potato (PGSC_DM_v4.03 genome assembly). (PDF 258 kb) Additional file 5: Table S2. LincRNA conservation analysis. (XLSX 32 kb) Additional file 6: Figure S2. Comparison of the 1113 lincRNA transcripts identified in the present study with potato lncRNAs available in the GreenC database. (PDF 105 kb) Additional file 7: Table S3. Differentially expressed lincRNA transcripts. (XLSX 231 kb) Additional file 8: Table S4. LincRNAs targeted by potato miRNAs. (XLS 30 kb) Additional file 9: Figure S3. RT-qPCR confirmation of five potato defense-related miRNAs in S. tuberosum cv BP1, computationally predicted to target some of the lincRNA transcripts. U6 snRNA was used as the reference gene. The fold changes of miRNAs at each time point were calculated relative to calibrator (control sample; 0 hpi). The experiments were done in triplicate. Error bars represent the fold change range calculated by 2^-(ΔΔCt±SD)^. (PDF 86 kb)
