# Supplementary figures and images for: Genome-wide identification of potato long intergenic noncoding RNAs responsive to Pectobacterium carotovorum subspecies brasiliense infection
Source: BMC Genomics. 2016 Aug 11;17:614. doi: 10.1186/s12864-016-2967-9 (PMC4982125; doi:10.1186/s12864-016-2967-9)

**A**

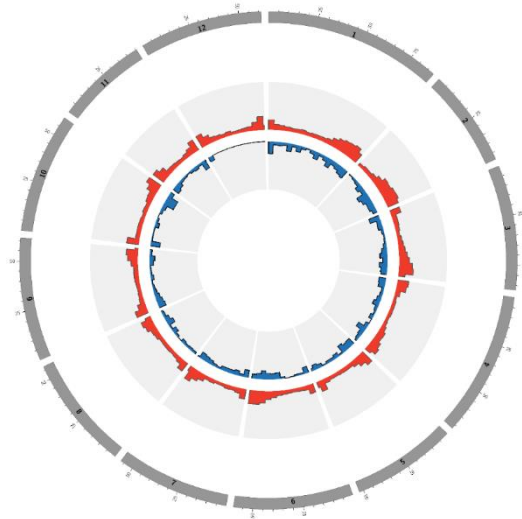

**B**

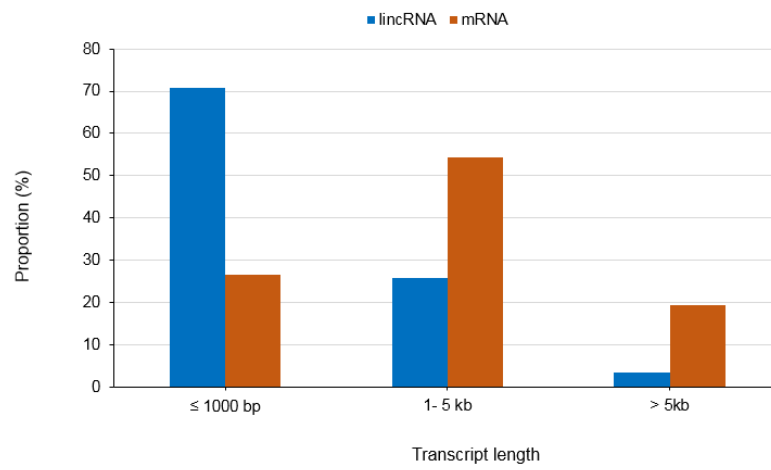

Supplement: Additional file 4: Figure S1. — A) Comparison of the genomic distribution of lincRNAs and protein-coding genes across the 12 potato chromosomes. The outer grey track represents the 12 potato chromosomes, with a scale (Mb) showing the length of each chromosome. The red histograms (second track with an outer orientation) and blue histograms (third track with inner orientation) represent the abundance and distribution of mRNA and lincRNAs, respectively, throughout the potato genome. The bin size (histogram width) = 5 Mbp). B) Comparison of LincRNA lengths to protein-coding mRNA transcripts in potato (PGSC_DM_v4.03 genome assembly). (PDF 258 kb) [file 12864_2016_2967_MOESM4_ESM.pdf]

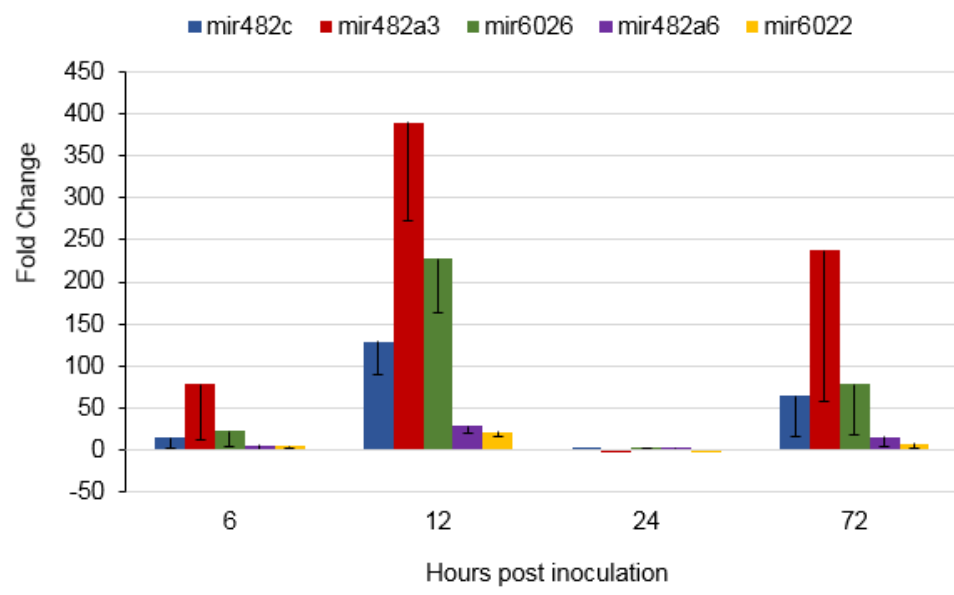

Supplement: Additional file 9: Figure S3. — RT-qPCR confirmation of five potato defense-related miRNAs in S. tuberosum cv BP1, computationally predicted to target some of the lincRNA transcripts. U6 snRNA was used as the reference gene. The fold changes of miRNAs at each time point were calculated relative to calibrator (control sample; 0 hpi). The experiments were done in triplicate. Error bars represent the fold change range calculated by 2-(ΔΔCt±SD). (PDF 86 kb) [file 12864_2016_2967_MOESM9_ESM.pdf]
