# Supplementary material for: Genome-wide identification of potato long intergenic noncoding RNAs responsive to Pectobacterium carotovorum subspecies brasiliense infection
Source: BMC Genomics. 2016 Aug 11;17:614. doi: 10.1186/s12864-016-2967-9 (PMC4982125; doi:10.1186/s12864-016-2967-9)
Supplement: Additional file 6: Figure S2. — Comparison of the 1113 lincRNA transcripts identified in the present study with potato lncRNAs available in the GreenC database. (PDF 105 kb) [file 12864_2016_2967_MOESM6_ESM.pdf]

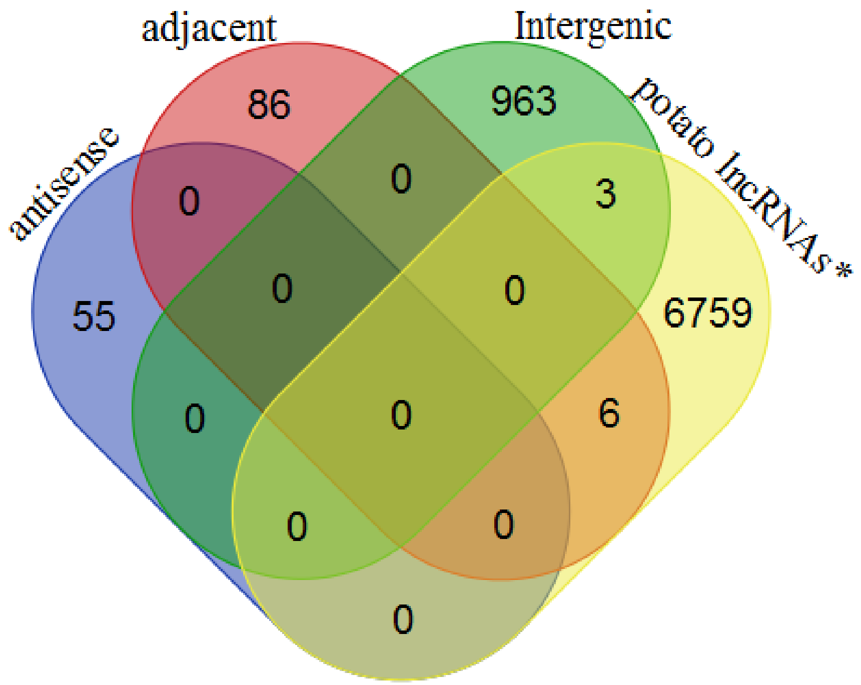

*Potato lncRNAs obtained from the GreenC database (http://greenc.sciencedesigners.com/wiki/Main_Page)
